# Effectiveness of rapid response teams in reducing intrahospital cardiac arrests and deaths: a systematic review and meta-analysis

**DOI:** 10.5935/0103-507X.20180049

**Published:** 2018

**Authors:** Hermano Alexandre Lima Rocha, Antônia Célia de Castro Alcântara, Sabrina Gabriele Maia Oliveira Rocha, Cristiana Maria Toscano

**Affiliations:** 1 Centro Universitário Unichristus - Fortaleza (CE), Brasil.; 2 Hospital Regional da Unimed Fortaleza - Fortaleza (CE), Brasil.; 3 Universidade Federal do Ceará - Fortaleza (CE), Brasil.; 4 Universidade Federal de Goiás - Goiânia (GO), Brasil.

**Keywords:** Patient care team, Rapid response teams, Mortality, Heart arrest, Quality of health care, Systematic review

## Abstract

**Objective:**

To evaluate the effectiveness of rapid response teams using early identification of clinical deterioration in reducing the occurrence of in-hospital mortality and cardiorespiratory arrest.

**Data sources:**

The MEDLINE, LILACS, Cochrane Library, Center for Reviews and Dissemination databases were searched.

**Study selection:**

We included studies that evaluated the effectiveness of rapid response teams in adult hospital units, published in English, Portuguese, or Spanish, from 2000 to 2016; systematic reviews, clinical trials, cohort studies, and prepost ecological studies were eligible for inclusion. The quality of studies was independently assessed by two researchers using the Newcastle-Ottawa, modified Jadad, and Assessment of Multiple Systematic Reviews scales.

**Data extractions:**

The results were synthesized and tabulated. When risk measures were reported by the authors of the included studies, we estimated effectiveness as 1-RR or 1-OR. In pre-post studies, we estimated effectiveness as the percent decrease in rates following the intervention.

**Results:**

Overall, 278 studies were identified, 256 of which were excluded after abstract evaluation, and two of which were excluded after full text evaluation. In the meta-analysis of the studies reporting mortality data, we calculated a risk ratio of 0.85 (95%CI 0.76 - 0.94); and for studies reporting cardiac arrest data the estimated risk ratio was 0.65 (95%CI 0.49 - 0.87). Evidence was assessed as low quality due to the high heterogeneity and risk of bias in primary studies.

**Conclusion:**

We conclude that rapid response teams may reduce in-hospital mortality and cardiac arrests, although the quality of evidence for both outcomes is low.

## INTRODUCTION

The quality of service provided to hospitalized patients deserves greater attention in tertiary hospitals.^(1)^ Several methods of measuring hospital quality are employed to assess services, including accreditation processes.^(2-4)^ One of the strategies suggested in the accreditation process that may improve quality of care and reduce hospital mortality is the implementation of rapid response teams (RRT), also known as emergency medical teams, code team/blue code teams, or cardiac arrest teams.

Rapid response teams are composed of health professionals dedicated exclusively to providing care to hospitalized patients identified as being at high risk for worsening prognoses. Rapid response teams are implemented with the aim of preventing cardiac arrest in patients admitted to hospital wards and, therefore, reducing in-hospital mortality.^(5)^

Rapid response team implementation was considered a priority intervention in the American "5 Million Lives" campaign.^(6)^ This campaign was implemented in 2004 by the Institute for Healthcare Improvement,^(4)^ with the objective of decreasing the number of deaths in the United States by 5,000,000 in two years. Since this campaign, the implementation of RRTs has been recommended by most accreditation agencies.^(6)^

A study conducted in three emergency hospitals in Australia showed that approximately 67% of deaths in hospitalized patients occurred in open ward units.^(7)^ It is estimated that patients who have cardiac arrests generally present with symptoms or clinical signs that predict the occurrence six to eight hours before the event.^(5)^ The most common signs of cardiac arrest among 66% of examined patients are desaturation and hypotension, findings that were verified in several studies conducted in hospitals with different conditions and structures.^(7-10)^

The idea of a RRT originated from trauma teams trained to recognize the signs of early clinical deterioration and rapidly respond to the needs of trauma patients, first introduced in Australia in 1989.^(11)^ Rapid response team composition often differs across hospitals. Some institutions have teams that comprise medical doctors, intensive care nurses, and physiotherapists,^(12,13)^ and in most hospitals, medical doctors serve as the RRT coordinators.^(14)^ Rapid response teams screen and treat inpatients with signs of clinical deterioration.^(15)^ The failure of the detection of this deterioration can reduce the effectiveness of RRTs.^(16)^ Additionally, RRTs were included as the fifth link in the chain of survival described in the Advanced Cardiac Life Support Subcommittee statement.^(17,18)^

The effectiveness of RRTs remains controversial because the available evidence regarding their impact is inconsistent.^(19-23)^ In a meta-analysis conducted by the Cochrane Collaboration in 2007, the effectiveness of RRTs could not be definitively concluded, mainly due to the number of studies using inappropriate methodology or having a low level of evidence.^(23)^

The results of another meta-analysis performed in 2010 demonstrated the effectiveness of RRTs, identifying a significant reduction in the number of cardiorespiratory arrests in adults (relative risk - RR = 0.66, 95% confidence intervals - 95%CI 0.54 - 0.80) but a nonsignificant reduction in mortality (RR = 0.96, 95%CI 0.84 - 1.09).^(24)^ The results of the most recent meta-analysis, which was conducted in 2015 and evaluated studies published up to 2013, indicated the presence of statistically significant reductions in mortality (13%) and cardiac arrests (35%).^(25)^ Subsequently, other studies evaluating RRT effectiveness were published,^(19,20,26-29)^ justifying the need for an updated meta-analysis on this subject.

Thus, we aimed to conduct a systematic review to examine the available scientific evidence examining the effectiveness of RRT in reducing hospital mortality and cardiac arrests. The outcomes studied were reductions in mortality and cardiac arrest occurrences among adult patients admitted to hospital wards.

## METHODS

### Data source and study selection

A systematic literature review was performed according to the PRISMA guidelines.^(30)^ The research question was developed using the PICOS strategy (Table 1).

**Table 1:** PICOS strategy to literature search

**P**opulation	Adult patients admitted by all causes in hospital open units
**I**ntervention	Rapid response team with early clinical deterioration identification systems
**C**omparison	Health services to patients in open units that do not use systems for early identification of clinical deterioration and without flow of triggering medical staff
**O**utcomes	Hospital mortality Cardiac arrest in open unit
**S**tudy design	Before-after ecological studies, clinical trials, cohort studies and meta-analyses

A search of the MEDLINE (by PubMed), Cochrane Library, Center for Reviews and Dissemination, and LILACS databases was conducted on February 1, 2016, to identify relevant literature; specific search strategies using the syntax and search engine of each database were used to obtain the highest possible sensitivity (Table 2). A manual search of the references of included studies was also conducted. The search was restricted by language (articles in Portuguese, English or Spanish) and date (articles that had been published since 2000).

**Table 2:** Article search strategies in electronic databases

Databases	List of terms
MEDLINE (Pubmed)	#1 ("Hospital Rapid Response Team"[Mesh]) AND "Hospital Mortality"[Mesh] #2 ("Hospital Rapid Response Team"[Mesh]) AND "Heart Arrest"[Mesh]
Cochrane Library	(tw:("rapid response team")) AND (tw:("mortality hospital")) (tw:("rapid response team")) AND (tw:("cardiac arrest"))
Centre for Reviews and Dissemination	"rapid response team" AND “mortality” "rapid response team" AND “cardiac arrest”
LILACS	(rapid response team) or "RAPID RESPONSE TEAM" [Palavras] and (mortality) or "MORTALITY, HOSPITAL" [Palavras] (rapid response team) or "RAPID RESPONSE TEAM" [Palavras] and "*PARADA CARDIO-RESPIRATORIA*" [Palavras]

We included studies that enrolled adults and assessed the effectiveness of RRT relative to no RRT in relation to hospital mortality or cardiac arrest in open hospital units. Only pre-post ecological studies, clinical trials, cohort studies, and meta-analyses that reported quantitative measures of either outcome were included.

Articles were independently selected by two researchers. Articles were initially selected based on the title and abstract. Then, duplicate articles and articles that did not meet the inclusion criteria were excluded. When disagreements occurred, the appraisers jointly reviewed the articles to reach a consensus. The full texts of the selected articles were obtained for comprehensive review. The quality of the articles was evaluated using the following tools: the Newcastle-Ottawa Scale (NOS) for cohort studies,^(31)^ the modified Jadad scale for clinical trials,^(32)^ and the Assessment of Multiple Systematic Reviews (AMSTAR) for systematic reviews.^(33)^

Two researchers also independently performed quality assessments, classifying the articles as good, medium, or poor quality according to the criteria of each scale. When disagreements occurred during quality evaluation, the evaluators jointly reviewed and discussed each article until a consensus was reached. Articles assessed as being of poor quality were excluded from the study.

We also considered the potential impact of novel research on our confidence in the effect estimates reported by the studies. To do so, the quality of evidence was assessed using the GRADE system. Evidence was classified as high (very unlikely that novel research will affect our confidence in the effect estimates); moderate (novel research may have a major impact on our confidence in the effect estimates); low (very likely that novel research may have a major impact on our confidence in the estimate); and too low (the validity of the effect estimates is uncertain).

### Data extraction

Information on the study design, population, outcome measures, results and limitations was extracted from each study.

For all studies, the primary association evaluated and extracted was the effect of RRTs on reducing the outcomes of interest. In cohort and case-control studies, this effect was reported as RR or odds ratio (OR). In pre-post studies, the effect was reported as rates during the periods before and after the intervention.

To allow comparison of the impact measures, we pooled the effectiveness estimates extracted from all studies. For cohort and case-control studies, effectiveness was presented as 1-OR or 1-RR. When possible, the effectiveness of pre-post studies was calculated as the percent decrease of the rates of interest following the intervention using the following formula: (preoccurrence rate - post occurrence rate)/preoccurrence rate x 100.

The results are presented for the comparisons of study design and type of outcome.

### Statistical analysis

Meta-analyses of primary study results were performed using the inverse variance method in random effects models to compensate for heterogeneity across studies, and the analyses were conducted using Review Manager 5.3 software.

## RESULTS

**Figure 1:**
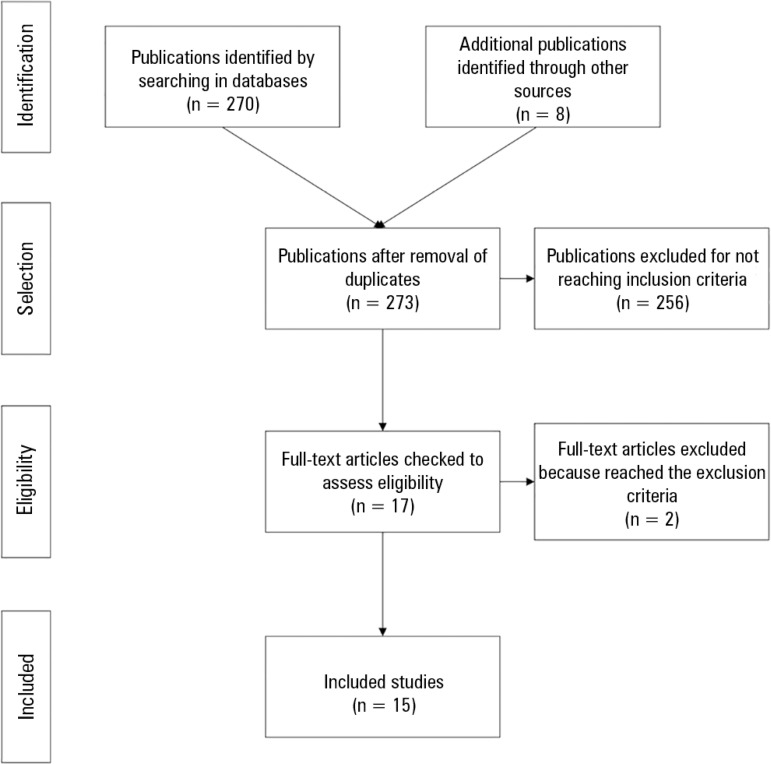
Diagram of evaluation and selection of articles found.

Overall, 278 articles were identified, five of which were duplicates and excluded. We evaluated the titles and abstracts of 273 articles, but 256 of these studies did not meet the inclusion criteria. Thus, 17 full text articles were evaluated, two of which were excluded because they did not meet the required quality standards (Figure 1). Finally, 15 articles published between 2000 and 2016 were included, including 2 clinical trials, 3 meta-analyses and 10 observational studies.

Table 3 presents the articles included in the review and the results of the initial quality evaluation, including the scores assigned using the NOS^(31)^ for observational studies, modified Jadad scale^(32)^ for clinical trials, and AMSTAR for systematic reviews. Methodological limitations were observed in most studies, but they did not appear to compromise the validity of their results and did not result in exclusion.

**Table 3:** Results of paired evaluation on the quality of the observational articles selected

Article	Checklist	Final conclusion in the scale
McGaughey et al.^(23)^	AMSTAR	All items were yes
Chan et al.^(24)^	AMSTAR	No explicit question does not present the record of meta-analysis
Maharaj et al.^(25)^	AMSTAR	All items were yes
Salvatierra et al.^(26)^	Jadad	8 points (inappropriate concealment)
Ludikhuize et al.^(29)^	NOS	7 stars (poor comparability)
Buist et al.^(34)^	Jadad	6 points (inappropriate concealment)
DeVita et al.^(35)^	NOS	7 stars (poor comparability)
Priestley et al.^(36)^	Jadad	5 points (inappropriate randomization and concealment)
Jones et al.^(37)^	Jadad	6 points (inappropriate concealment)
Hillman et al.^(38)^	Jadad	6 points (inappropriate concealment)
Dacey et al.^(39)^	Jadad	8 points (inappropriate concealment)
Chan et al.^(40)^	Jadad	8 points (inappropriate concealment)
Konrad et al.^(41)^	Jadad	8 points (inappropriate concealment)
Beitler et al.^(42)^	NOS	7 stars (poor comparability)
Gonçales, et al.^(43)^	Jadad	8 points (inappropriate concealment)

The study population assessed, study design employed, outcomes evaluated, effectiveness estimates and their corresponding 95%CI and p-values, and level of evidence classification are described for each study included in this review (Table 4).

**Table 4:** Results of clinical trials, before-after ecological and cohort studies included in the systematic review

Studies	Study design/population	Outcome	Results	Study limitations	Effectiveness (%)	Quality of evidence
McGaughey et al.^(23)^	Systematic review Studies published between 1996 and June 2006 Outcome: mortality Intervention: Introduction of early warning scores by RRT	Mortality	Reduction in mortality	Only two studies were examined; did not conduct economic analyses	Reduction in mortality	Moderate
Chan et al.^(24)^	Systematic review and meta-analysis Studies published between 01 January 1950 and 31 November 2008 Outcome: mortality and cardiopulmonary arrest Intervention: Introduction of RRT	Mortality Cardiac arrest	RR 0.96 (95%CI 0.84 - 1.09) RR 0.66 (95%CI 0.54 - 0.80)	Did not analyze data at the individual level; academic centers were used in most studies reviewed	4% (95%CI -9 - 16) 0.34 (95%CI 0.20 - 0.46)	Moderate
Maharaj et al.^(25)^	Systematic review and meta-analysis Studies published between 01 January 1990 and 31 November 2013 Outcome: mortality and cardiopulmonary arrest Intervention: Introduction of RRT	Mortality Cardiac arrest	RR 0.87 (95%CI 0.81 - 0.95) (p < 0.001) RR 0.65 (95%CI 0.61 - 0.70) (p < 0.001)	Did not analyze data at the individual level	13% (95%CI 5 - 19) 35% (95%CI 30 - 39)	Moderate
Salvatierra et al.^(26)^	Before-after study Adult patients Intervention: Introduction of medical emergency teams in 10 hospitals in Washington over 31 months, 235344 patients Control: 235718 patients before intervention	Mortality	RR 0.76 (95%CI 0.72 - 0.80)	Used historical controls	24% (95%CI 20 - 28)	Low
Ludikhuize et al.^(29)^	Before-after study Adult patients Intervention: The implementation of RRS was divided into two phases. First, the MEWS (Modified Early Warning Score) and the SBAR communication tools were administered; then after 7 months, RRTs were implemented in 12 Dutch hospitals, 29560 admissions Control: 28298 admissions	Mortality Cardiac arrest	OR 0.80 (95%CI 0.64 - 1.00) OR 0.60 (95%CI 0.39 - 0.93)	Used historical controls	20% (95%CI 0 - 36) 40% (95%CI 7 - 61)	Low
Buist et al.^(34)^	Before-after study Adult patients in American hospitals with 300 beds Intervention: Introduction of rapid response teams Controls: historical, 19317 admissions	Cardiac arrest	OR 0.5 (95%CI 0.35 - 0.73)	Used historical controls	50% (95%CI 27 - 65)	Low
DeVita et al.^(35)^	Retrospective analysis of outcomes Adult patients in American hospitals with 622 beds Intervention: Introduction of objective criteria medical emergency team activation	Cardiac arrest	6.5 to 5.4/1000	Observational study; retrospective analysis with confounders that were difficult to control for	16%	Moderate
Priestley et al.^(36)^	Clinical trial randomized by wards 16 wards in an 800-bed general hospital in England Outcome: mortality Intervention: Introduction of critical care service in wards	Mortality	OR 0.52 (95%CI 0.32 - 0.85)	Few hospitals participated; Hawthorne effect; contamination of controls; problems with data collection	48% (95%CI 15 - 68)	Moderate
Jones et al.^(37)^	Before-after study, analysis of three periods Adult patients in an Australian hospital with 400 beds Intervention: Introduction of medical emergency teams Controls: historical, 16246 admissions	Cardiac arrest	OR 0.47 (95%CI 0.35 - 0.62)	Used historical controls; not randomized or blinded; only one hospital was evaluated; cardiac arrest reduction mechanism was not revealed	53% (95%CI 38 - 65)	Low
Hillman et al.^(38)^	Randomized trial Adult patients in 23 Australian hospitals Outcome: cardiopulmonary arrest and unexpected death Intervention: introduction of medical emergency teams Control: No introduction of medical emergency team	Mortality Cardiac arrest	OR 1.03 (95%CI 0.84 - 1.28) OR 0.94 (95%CI 0.79 - 1.13)	Variations found between hospitals were higher than was anticipated by the researchers	-3% (95%CI -28 - 16) 6% (95%CI -13 - 21)	Moderate
Dacey et al.^(39)^	Before-after study Adult patients in American hospitals with 350 beds Intervention: Introduction of rapid response teams Controls: historical	Mortality Cardiac arrest	2.82 to 2.35/100 hospitalizations (p < 0.001) 7.6 to 3.0/1000 hospitalizations (p < 0.001)	Not randomized; Hawthorne effect	16% (p < 0.001) 60% (p < 0.001)	Low
Chan et al.^(40)^	Prospective cohort Adult patients in an American hospital with 404 beds Intervention: Introduction of rapid response teams Controls: historical, 24193 admissions	Mortality	OR 0.95 (95%CI 0.81 - 1.11)	Used historical controls, but adjusted for temporal trends; weak statistical power to identify differences in mortality	5% (95%CI -11 - 19)	Low
Konrad et al.^(41)^	Before-after study Adult patients Intervention: Introduction of medical emergency teams, 73825 patients Control: 203892 patients before intervention	Mortality Cardiac arrest	OR 0.90 (95%CI 0.84 - 0.97) OR 0.74 (95%CI 0.55 - 0.98)	Used historical controls; delays in the team drive were not evaluated	10% (95%CI 3 - 16) 26% (95%CI 2 - 45)	Low
Beitler et al.^(42)^	Prospective cohort Adult patients in American hospitals with 809 beds Intervention: Introduction of rapid response teams Controls: historical, 77021 patients	Mortality Cardiac arrest	RR 0.82 (95%CI 0.69 - 0.98) RR 0.49 (95%CI 0.39 - 0.61)	Used historical controls, but adjusted for temporal trends	18% (95%CI 2 - 31) 51% (95%CI 39 - 61)	Low
Gonçales et al.^(43)^	Before-after study Adult patients in Brazilian hospitals with 477 beds Intervention: Introduction of medical emergency teams Control: patients attended over the 19 months before intervention	Mortality Cardiac arrest	14.34/1000 after intervention 16.27 before (p < 0.001) 1.69/1000 hospitalizations 3.54 before (p < 0.001)	Used historical controls; delays in the team drive were not evaluated	11% (p < 0.001) 52% (p < 0.001)	Low

RR - relative risk; 95%CI - confidence intervals; RRT - rapid response teams; MEWS - Modified Early Warning Score; SBAR - situation, background, assessment, recommendation.

A total of 12 studies evaluated mortality. Nine of these studies yielded results indicating that RRTs are associated with a significant reduction in mortality, with estimates varying from 10 - 48%.^(25,26,29,34,36,41-43)^ The three remaining studies did not find RRTs to be effective in achieving reduced mortality.^(24,38,40)^ Of the three meta-analyses included, two reported no significant reduction in mortality.^(24,38)^ However, the most recent meta-analysis conducted in 2015 indicated a statistically significant reduction (Table 4).

Eleven studies considered the occurrence of cardiopulmonary arrests. Nine of these studies, including two meta-analyses, presented results indicating that RRTs are associated with a significant reduction in cardiopulmonary arrest occurrence, with ORs ranging between 0.47 and 0.74.^(25,29,35-37,39,41-43)^ The remaining two studies did not find RRTs to be effective in reducing cardiopulmonary arrest (Table 4).^(35,38)^

The most recent meta-analysis, which was conducted in 2015 and included studies published until 2013, reported the following pooled measures of RRT effectiveness: 13% for mortality (95%CI 5 - 19) and 35% for cardiopulmonary arrest (95% CI 30 - 39). Two studies that were conducted in 2014 and 2015 and not included in that meta-analysis also reported a significant reduction in mortality, with an RR of 0.76^(26)^ and OR of 0.80.^(29)^ In Brazil, RRTs have been found to be associated with significant reductions in the occurrence of mortality (11%) and cardiac arrest (52%) (Table 4).^(43)^

**Figure 2:**
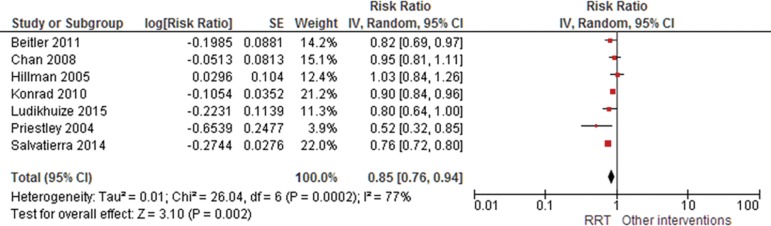
Forest plot of the effectiveness of rapid response teams in mortality prevention.

**Figure 3:**
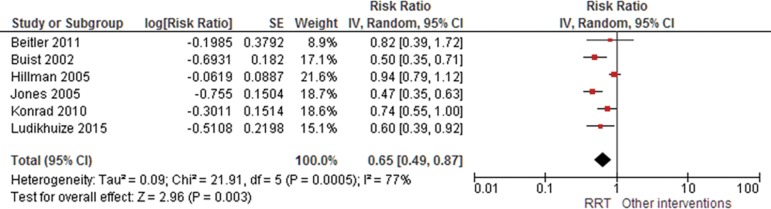
Forest plot of the effectiveness of rapid response teams in cardiac arrest prevention.

The results of the meta-analysis of studies reporting mortality suggested that RRTs demonstrated a protective effect, with a risk ratio of 0.85 (95%CI 0.76 - 0.94); similar results were identified for the occurrence of cardiac arrest (RR 0.65; 95%CI 0.49 - 0.87). Significant heterogeneity was observed (Figures 2 and 3). Evidence was assessed as low quality by the GRADE system due to high heterogeneity and risk of bias in primary studies.

## DISCUSSION

This systematic review found that the implementation of RRT can be issued as a B level recommendation because most studies have shown that teams effectively reduce in-hospital mortality and the occurrence of cardiac arrest in adults in open hospital units. Findings from the meta-analysis suggests that RRTs are associated with a 15% reduction in mortality.

Although the evidence supports only a B level of recommendation, it is important to consider ethical issues that may derail the provision of results with the highest level of evidence. The ethics of conducting studies to evaluate the effectiveness of RRTs may be questionable, as one group of patients receives the intervention, while the other is deprived of it. This complicates the use of control groups, the randomized allocation of the intervention, and the blinding of subjects to their received intervention. However, since it is a controversial issue, a clinical trial may be performed.

In an assessment of the optimal epidemiological design for evaluating health service quality, observational cohorts were identified as one of the best possible approaches available.^(44)^ This is why many studies utilized a pre-post design, at times using historical controls, which reduced the strength of the provided evidence. Thus, we believe that the evidence presented in this study may be the optimal way to assess the effectiveness of RRTs.

Nine of the fifteen evaluated studies found a significant reduction in mortality following the implementation of RRT, including a study and a meta-analysis that were recently published in 2015.^(25,26,29,36,39,41-43)^ For cardiorespiratory arrest, nine of the eleven studies reporting this outcome also indicated satisfactory results, showing a statistically significant reduction in cardiorespiratory arrest when hospitals implemented RRTs.^(25,29,34,37,39,41-43)^

The heterogeneity of the results is due in part to the different settings in which each study was performed, as well as to the different designs that each study used and the number of patients evaluated. Furthermore, the composition of teams in each study and the manner detecting clinical deterioration were not strictly the same, although sufficiently comparable.

Some previous studies have reported on the implementation of RRTs in Brazil.^(5,43,45,46)^ A large private tertiary hospital in São Paulo that instituted an RRT in 2005 evaluated the impact of this implementation. That study demonstrated that RRT implementation was associated with a significant reduction in the rates of cardiorespiratory arrest (from 3.54 to 1.69 per 1,000 discharges) and in-hospital mortality (16.27 to 14.34 deaths per 1,000 discharges).^(43)^ No official data are available regarding the number of Brazilian hospitals in which RRTs are currently implemented.

Other health technology assessment databases were searched for recommendations on the use of RRT, including the databases of the National Institute for Clinical Excellence and Health, National Institute for Health Research Health Technology Assessment Programme, Canadian Agency for Drugs and Technologies in Health, and *Agencias y Unidades de Evaluación de Tecnologías Sanitarias*, but no recommendations were identified regarding the use of RRTs. Therefore, health technology assessment agencies have not yet established recommendations on the use of RRTs in the countries in which they are located, perhaps because of the lack of evidence provided thus far.

One of this study's limitations was the heterogeneous nature of the hospitals in which RRT have been assessed. Many if the included studies used historical controls, the implications of which have been previously mentioned and discussed. In addition, randomized controlled intervention trials and blinded assessments of the effectiveness of the intervention were not identified. Finally, gray literature was not searched, and language was restricted.

## CONCLUSION

We conclude that rapid response teams may reduce in-hospital mortality and cardiac arrest, although the quality of evidence for both outcomes is low.
